# Activating transcription factor 4 (ATF4) modulates post-synaptic development and dendritic spine morphology

**DOI:** 10.3389/fncel.2014.00177

**Published:** 2014-06-30

**Authors:** Jin Liu, Silvia Pasini, Michael L. Shelanski, Lloyd A. Greene

**Affiliations:** Department of Pathology and Cell Biology, Columbia University Medical CenterNew York, NY, USA

**Keywords:** ATF4, post-synaptic development, mushroom spines, filopodia, Cdc42

## Abstract

The ubiquitously expressed activating transcription factor 4 (ATF4) has been variably reported to either promote or inhibit neuronal plasticity and memory. However, the potential cellular bases for these and other actions of ATF4 in brain are not well-defined. In this report, we focus on ATF4's role in post-synaptic synapse development and dendritic spine morphology. shRNA-mediated silencing of ATF4 significantly reduces the densities of PSD-95 and GluR1 puncta (presumed markers of excitatory synapses) in long-term cultures of cortical and hippocampal neurons. ATF4 knockdown also decreases the density of mushroom spines and increases formation of abnormally-long dendritic filopodia in such cultures. *In vivo* knockdown of ATF4 in adult mouse hippocampal neurons also reduces mushroom spine density. In contrast, ATF4 over-expression does not affect the densities of PSD-95 puncta or mushrooom spines. Regulation of synaptic puncta and spine densities by ATF4 requires its transcriptional activity and is mediated at least in part by indirectly controlling the stability and expression of the total and active forms of the actin regulatory protein Cdc42. In support of such a mechanism, ATF4 silencing decreases the half-life of Cdc42 in cultured cortical neurons from 31.5 to 18.5 h while knockdown of Cdc42, like ATF4 knockdown, reduces the densities of mushroom spines and PSD-95 puncta. Thus, ATF4 appears to participate in neuronal development and plasticity by regulating the post-synaptic development of synapses and dendritic mushroom spines via a mechanism that includes regulation of Cdc42 levels.

## Introduction

Activating transcription factor 4 (ATF4), also known as cAMP response element binding protein 2 (CREB-2), belongs to the ATF/CREB transcription factor family of basic leucine zipper domain proteins (Hai and Hartman, 2001). It may either activate or inhibit gene transcription. ATF4 is ubiquitously expressed and participates in a wide range of activities (Ameri and Harris, 2008). For instance, ATF4 is involved in development of lens, bone, and vas deferens (Tanaka et al., 1998; Masuoka and Townes, 2002; Fischer et al., 2004; Yang et al., 2004). ATF4 is also induced by multiple cellular stresses and under stress conditions, can mediate either cell death or survival in a context-dependent manner (Ameri and Harris, 2008; Ye and Koumenis, 2009).

As in other tissues, ATF4 plays multiple roles in brain. It is induced in neurons by stress and promotes cell survival or death (Lange et al., 2008; Ogawa et al., 2008; Galehdar et al., 2010; Sun et al., 2013). ATF4 is upregulated in neurodegenerative disorders including Parkinson's and Alzheimer's diseases (Lewerenz and Maher, 2009; Yoon et al., 2012; Sun et al., 2013), but its role(s) in these maladies remains undefined. ATF4 is also decreased in the frontal cortex of schizophrenia patients (Trinh et al., 2012) and its binding to DISC1 (disrupted-in-schizophrenia 1) is compromised by schizophrenia-associated DISC1 mutations (Morris et al., 2003). Additionally, ATF4 is induced in the nucleus accumbens and dorsal striatum by restraint or amphetamine, while ATF4 overexpression in the nucleus accumbens affects emotional reactivity (Green et al., 2008).

A body of work also implicates ATF4 in regulation of synaptic plasticity and memory. In *Aplysia*, *Ap*CREB-2, which is homologous to ATF4, represses long-term facilitation (Bartsch et al., 1995; Lee et al., 2003). Several studies in mice have also been interpreted to indicate that ATF4 impairs LTP and memory consolidation (Chen et al., 2003; Costa-Mattioli et al., 2005, 2007; Jiang et al., 2010). On the other hand, additional reports suggest that ATF4 is required for behavioral flexibility (Trinh et al., 2012) and consolidation of object recognition memory (Ill-Raga et al., 2013) and enhances memory for fear extinction (Wei et al., 2012). The apparently diverging views of ATF4's role in learning and memory may reflect the use of indirect and likely non-selective means that have been used to experimentally affect ATF4 expression.

Interpretation of the many potential, and possibly conflicting, roles of ATF4 in neurons requires a better understanding of its actions at the cellular level as well as direct and specific means to manipulate its expression. Here, we have developed lentiviral vectors to directly regulate ATF4 expression in neurons and have used these to examine the roles of ATF4 in synapse formation and dendritic spine morphology. We find that knockdown of endogenous ATF4 in cultured neurons decreases the density of post-synaptic markers and reduces the density of dendritic mushroom spines. In addition, knockdown of ATF4 in the adult mouse hippocampus decreases mushroom spine density. The mechanism of these effects appears to be mediated in part by reduced stability of the cytoskeletal-regulatory protein Cdc42.

## Materials and methods

### DNA constructs and real-time quantitative PCR

For lentiviral constructs, all shRNAs were generated in the pLVTHM vector (Addgene), which contains the H1 promoter for shRNA production and a separate promoter-driven eGFP to indicate transduction. All ATF4 overexpression lentiviral constructs were made in pWPI vector (Addgene), which contains an EF-1α promoter for target gene expression and an internal ribosomal entry site (IRES) for eGFP expression as a reporter. Lenti-shATF4 was generated by using the following oligo DNA pair: 5′-CGCGTGCCTGACTCTGCTGCTTATTTCAAGAGAATAAGCAGCAGAGTCAGGCTTTTTTA-3′ and 5′-CGCGTAAAAAGCCTGACTCTGCTGCTTATTCTCTTGAAATAAGCAGCAGAGTCAGGCA-3′. The oligo DNA pairs to generate two shRNA controls for shATF4 were as follows: control-1 5′-CGCGTGCCAGATTCAGCGGCCTACATTTCAAGAGAATGTAGGCCGCTGAATCTGGCTTTTTTA-3′ and 5′-CGCGTAAAAAAGCCAGATTCAGCGGCCTACATTCTCTTGAAATGTAGGCCGCTGAATCTGGCA-3′; control-2 5′-CGCGTCACAGCCCTTCCACCTCCATTCAAGAGATGGAGGTGGAAGGGCTGTGTTTTTTA-3′ and 5′-CGCGTAAAAAACACAGCCCTTCCACCTCCATCTCTTGAATGGAGGTGGAAGGGCTGTGA-3′. The over-expression construct lenti-ATF4 was generated by inserting the rat ATF4 cDNA into the pWPI vector using PCR primer pair: 5′-ATGACCGAGATGAGCTTCC-3′ and 5′-TTAAGGAACTCTCTTCTTC-3′. Lenti-ATF4add, which is resistant to shATF4, was derived from lenti-ATF4 by introducing point mutations into the recognized site of shATF4 (CCTGACTCTGCTGCTTAT to CCAGAGTCAGCTGCTTAC) using the QuickChange Site-directed Mutagenesis kit (Stratagene). These point mutations do not change amino acid coding in the sequence. Dominant-negative ATF4 (lenti-shATF4 add/mut) was derived from shATF4add by introducing point mutations into the DNA binding site (292RYRQKKR298 to 292GYLEAAA298) as descripted previously (Luo et al., 2003).

The lentiviral construct for RhoA silencing (lenti-shRhoA) was derived from the siRNA sequence 5′-GCCACUUAAUGUAUGUUAC-3′ (Otsuka et al., 2011) by using the pLVTHM vector. A scrambled control shRNA (lenti-shRhoAscr) was generated using the same vector and the following oligo DNA pair: 5′-CGCGTGGCAAATCTTCTAGTCTATTTCAAGAGAATAGACTAGAAGATTTGCCTTTTTTA-3′ and 5′-CGCGTAAAAAAGGCAAATCTTCTAGTCTATTCTCTTGAAATAGACTAGAAGATTTGCCA-3′. Lenti-shCdc42 was generated according to a published siRNA sequence 5′-GAUAACUCACCACUGUCCATT-3′ (Deroanne et al., 2005). A scrambled shRNA (lenti-shCdc42scr) was generated by using the following oligo DNA pair: 5′-CGCGTGTCCAACGTCCATATACCATTCAAGAGATGGTATATGGACGTTGGACTTTTTTA-3′ and 5′-CGCGTAAAAAAGTCCAACGTCCATATACCATCTCTTGAATGGTATATGGACGTTGGACA-3′.

To assess the level of endogenous Cdc42 mRNA in cultured neurons, total RNA was isolated at 4, 8, 12 days after infection with lenti-shATF4 or control by using RNeasy Mini Kit (Qiagen). Reverse transcription was performed by using the First-strand cDNA Synthesis Kit (Origene) following the manufacturer's instructions. Quantitative PCR was carried out in a “realplex2” machine (Eppendorf) by using the following primer pair: 5′-GCTTGTCGGGACCCAAATTG-3′ and 5′-ACACCTGCGGCTCTTCTTCG-3′. PCR with a primer for α-tubulin was used to normalize RNA levels.

### Lentivirus preparation

For lentivirus preparation for *in vitro* experiments, the second generation packaging system (which generates replication-deficient lentivirus) was used for all experiments (Zufferey et al., 1997). Packaging vectors psPAX2 and pMD2.G were obtained from Addgene. Briefly, lentiviral constructs for shRNA or overexpression were co-transfected with the packaging vectors into HEK293T cells with calcium phosphate. Supernatants containing virus were collected 24 h and 48 h after transfection. After centrifugation at 1000 rpm for 10 min, the supernatants were passed through a 0.45 μm PDVF filter unit (Nalgene). The viruses were concentrated 20–30x by centrifugation in an Amicon Ultra centrifugal filter (100 K) (Millipore) following the manufacturer's instructions. Viruses were aliquoted and stored at −80°C. Viral titers ranged from 1–5 × 10^6^ infectious units/μl. For *in vivo* experiments, virus preparations from above were further concentrated by ultracentrifuge at 50,000 rpm (Beckman TL-100 Ultracentrifuge) at 4°C for 1 h. Pellets containing virus were resuspended in PBS. Titers varied between 10^7^ and 10^9^ infectious units/μl.

### Cell culture, transfection, and infection

HEK293T cells were purchased from the ATCC and maintained in DMEM medium (Invitrogen) containing 10% FBS (Hyclone). For virus preparation, the calcium phosphate method was used for transfection.

Primary hippocampal and cortical cultures were prepared as previously described (Lesuisse and Martin, 2002) with slight modifications. Briefly, hippocampi or cortices from E18 rat embryos were collected, pooled, and plated on poly-D-lysine (Sigma) treated plates or coverslips in 12-well plates at a density of 3 × 10^5^/well. For cell staining experiments, neurons were cultured at a low density (3 × 10^4^/well) on cover glasses and maintained in conditioned medium (from regular density cultures). Neurons were maintained in Neurobasal medium (Invitrogen) supplemented with 2% B-27 (Invitrogen) and 0.5 mM glutamine (Invitrogen). Half of the culture medium was changed every 3 days after plating. For spine analysis experiments, lentiviruses were added to the cultures on day 5–6 *in vitro* and the cultures were fixed on day 18–19 *in vitro*.

### Antibodies, western blotting, immunocytochemistry, and diolistic labeling

For Western immunoblotting analysis, infected neurons were collected in 1 × LDS loading buffer (Invitrogen) and boiled for 10 min. Proteins were separated by electrophoresis in 12% NuPAGE gels (Invitrogen). To better detect ATF4, the gels were run a longer time (80–100 min) to separate ATF4 from a closely migrating non-specific band. The following primary antibodies were used: rabbit anti-ATF4 (1:250, PRF&L), mouse anti-RhoA (1:1000; Cytoskeleton), mouse anti-RhoA-GTP (1:500; Santa Cruz), mouse anti-Rac1 (1:5000; Millipore), mouse anti-Cdc42 (1:500; BD), rabbit anti-pLIMK1/2 (1:500; Santa Cruz), and mouse anti-GAPDH (1:2000; Imgenex).

Immunocytochemistry was performed on the primary hippocampal neurons cultured on 15-mm cover glasses. Neurons were fixed with 4% PFA for 15 min and blocked with 5% BSA. The following primary antibodies were used: rabbit anti-GluR1 (1:200; Cell Signaling), rabbit anti-PSD-95 (1:200; Cell Signaling).

For DiOlistic labeling, the Helios gene gun system (Bio-Rad) was used according to the manufacturer's instructions. Tungsten particles (1.1 μm, Bio-Rad) coated with Dil (Invitrogen), which defines the neuronal architecture in red, were delivered into fixed neurons on coverslips or brain sections. Coverslips were mounted in ProLong Gold antifade reagent (Invitrogen) and imaged the next day.

### Microscopy and image analysis

For cultured neuron spine morphology analysis, all images were captured on a Zeiss MLS 510 Meta confocal microscope with a 100×/1.3 NA oil-immersion objective. Consecutive Z-stacks were collected. PSD-95 and GluR1 puncta were measured by means of the NIH ImageJ program using the particle analysis plugin. Analysis of spine density and morphology was performed semi-automatically by using NeuronStudio software (Rodriguez et al., 2003). Mushroom spines were defined as short spines with a bulbous head (maximum head diameter ≥0.5 μm). Stubby spines were defined as short spines (≤0.3 μm) without heads. Thin spines were defined as long spines with a neck (head/neck diameter ratio ≥1.2) and a small head (<0.5 μm). Filopodia were defined as protrusions of dendrite without a distinctive head. 10 neurons were randomly picked up from each group in each experiment and 3–5 basal dendrites from each cell were analyzed. To avoid user bias, imaging and analysis were conducted in a blinded manner. Comparison of data and calculation of *p*-values were performed by using Student's paired two-tailed *t*-test. For these tests, we compared values from cultures treated with either control or experimental virus and treated these as a “pair.” Data include the total number of independent repeats performed per experiment.

For spine analysis of brain sections, GFP positive and DiOlistically labeled neurons were imaged at high magnification (60× oil-immersion objective) using Confocal Microscopy (Nikon). Ten images for each sample were taken using the Z-stack at 0.2 μm intervals to cover the full depth of the dendritic arbor. Analysis of spine density and morphology was performed automatically by using NeuronStudio software. To compare the averages between two groups, the unpaired two-tailed Student's *t*-test was used.

### CDC42-GTP, RAC1-GTP pull-down and CDC42 protein turnover analysis

To characterize the active “GTP-bound” forms of Rac and Cdc42, pull-down experiments were performed in the virus infected cortical neurons. Briefly, cells were lysed in RIPA buffer and centrifuged at 15,000 rpm for 10 min at 4°C. Supernatants were mixed with PAK-GST beads (Cytoskeleton) following the manufacturer's instructions. The levels of Rac-GTP and Cdc42-GTP were detected by Western immunoblotting using anti-Rac1 and anti-Cdc42 antibodies.

To determine the effect of ATF4 knockdown on Cdc42 protein half-life, primary cortical neurons were infected with shATF4 or control lentivirus on day 5–7 *in vitro*. One week after infection, cells were treated with 10 μM cycloheximide (Sigma) for 0–30 h. Cell lysates were collected and subjected to Western immunoblotting analysis to assess the relative levels of Cdc42 expression. The relative levels of Cdc42 remaining after the indicated times (0 time = 100; values = means from 4 independent experiments) were expressed on a ln2 scale and the half-life calculated by least-squares analysis.

### Animal injection and immunohistochemistry

For lentivirus injection experiments, 3-month old C57BL/6 male mice (Jackson Laboratory) were used. All the animal studies were performed according to protocols approved by Columbia University and the Institutional Animal Care and Use Committee. Briefly, mice were anesthetized and stereotactically injected with 2 μl of viral preparation through a 31G needle attached to a 50 μm Hamilton syringe at a speed of 0.5 μl/min over a period of 4 min. The coordinates with respect to bregma were 2.45 mm posterior, 1.8 mm lateral, and 2 mm deep from inner surface of the skull to injection point.

Six weeks after the lentivirus injection, mice were deeply anesthetized and fixed with 4% paraformaldehyde (PFA) by transcardial perfusion. Brains were removed and post-fixed overnight at 4°C in 4% PFA. The brains were sectioned coronally (200 μm) using a vibratome (Leica VT1200S). Brain sections were stained for GFP (1:1000; Invitrogen) to clearly visualize the infected cells, because in some cases the fluorescence of GFP itself was too weak to detect all infected cells and processes.

## Results

### ATF4 knockdown reduces the density of dendritic PSD95 and GLuR1 puncta in cultures of hippocampal neurons

**Figure 1:**
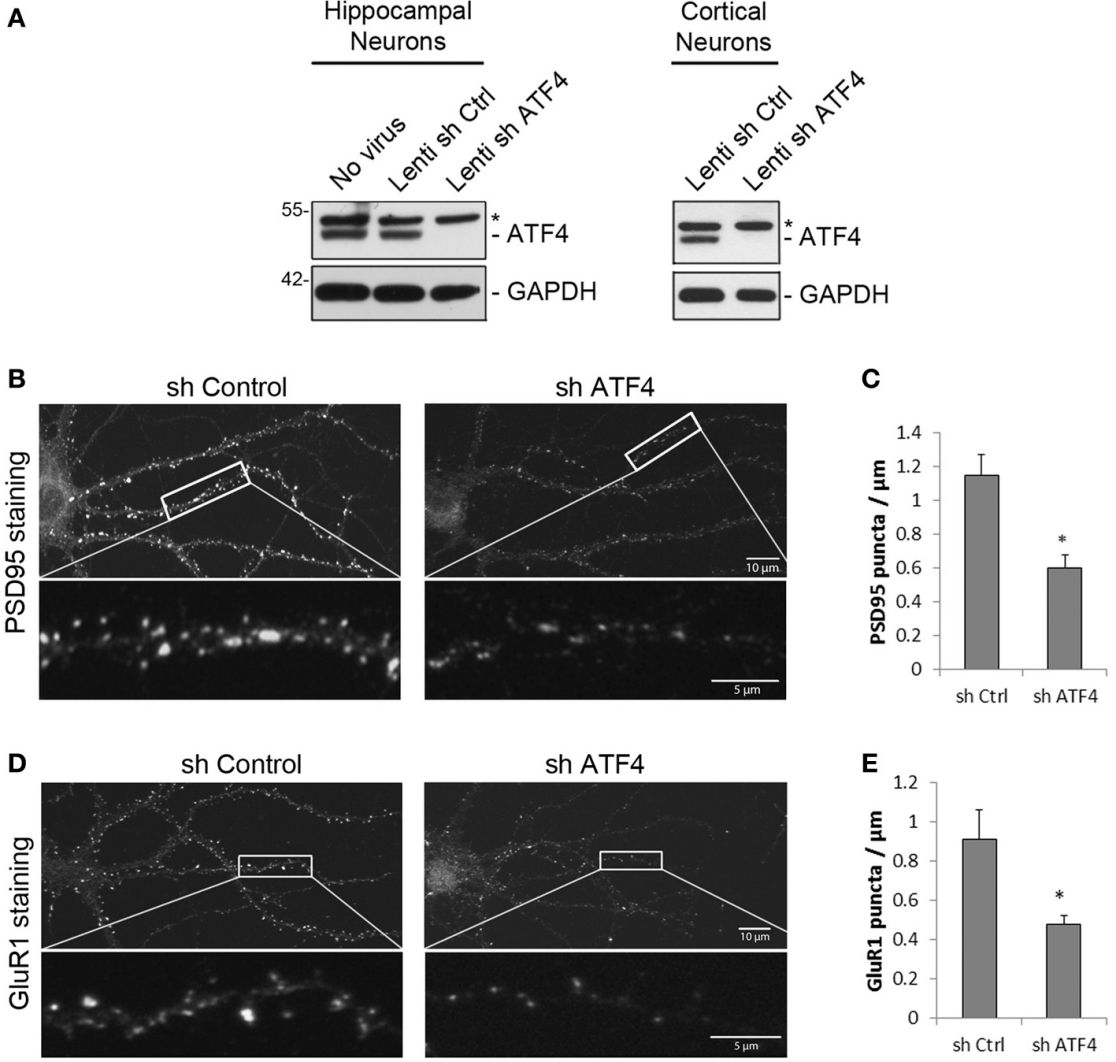
**ATF4 affects the density of dendritic PSD95 and GluR1 puncta in rat primary hippocampal neurons. (A)** shATF4 lentivirus knocks down endogenous ATF4. Cultured neurons were infected with lenti-shATF4 or lenti-Ctrl on 7 DIV (days *in vitro*) and harvested at 14 DIV for Western immunoblotting. ^*^Indicates a non-specific band on the Western blot. **(B–E)** ATF4 knockdown reduces the density of PSD95 and GluR1 puncta. Cultured hippocampal neurons were infected with viruses at 6 DIV and stained for PSD95 or GluR1 at 19 DIV. Quantifications of puncta density are in **(C,E)**. Data from 3 independent experiments, 12 neurons/group, ^*^p < 0.05.

**Figure 2:**
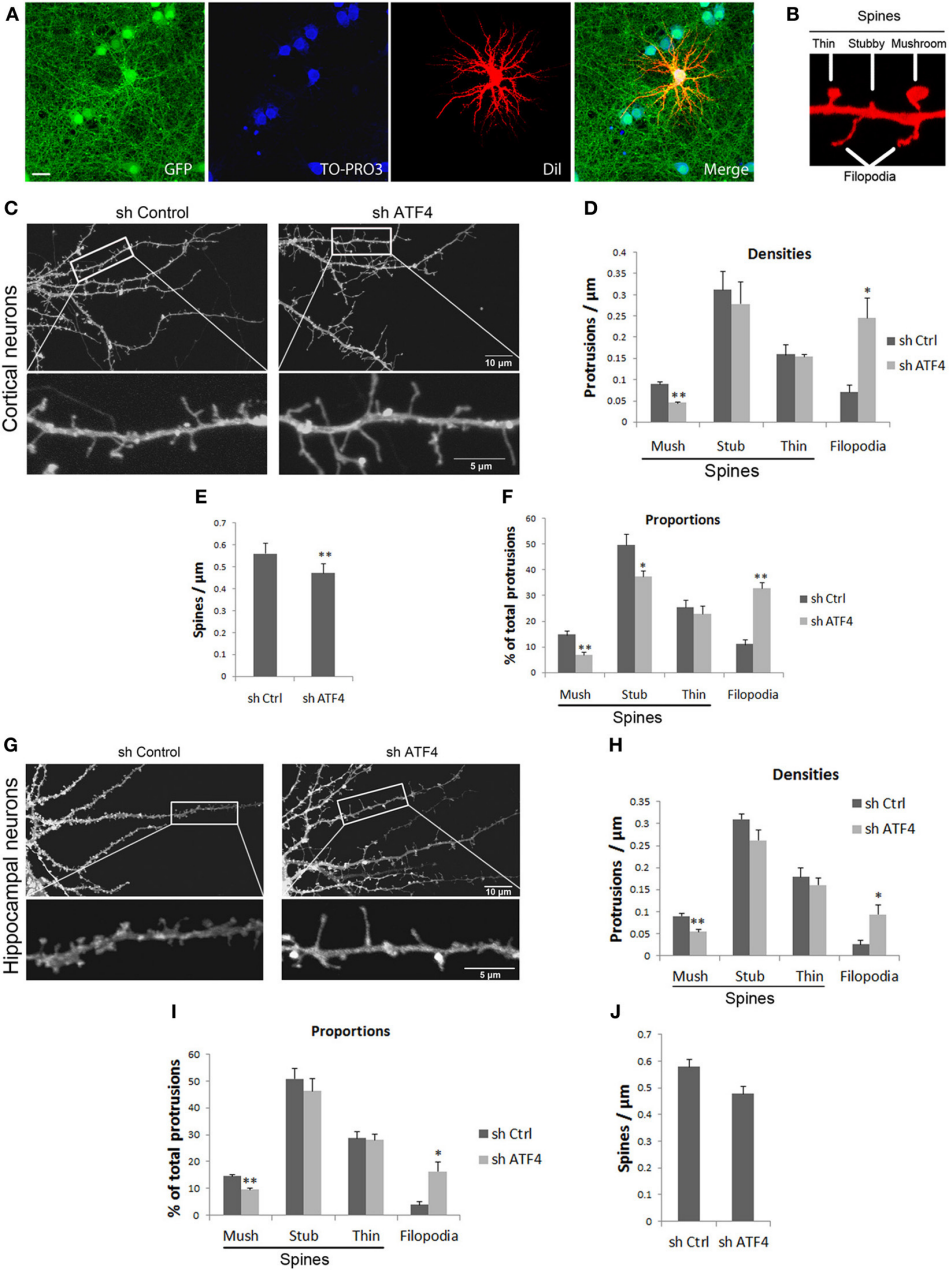
**Knockdown of ATF4 decreases the density of mushroom spines and increases filolopia in cultures of primary neurons. (A)** Neuron infection and labeling for spine analysis. Cultured cortical neurons were infected with lentivirus at 6 DIV and fixed at 18 DIV. Infected cells (green) were randomly filled with Dil (red) by DiOlistic labeling and analyzed by confocal microscopy. **(B)** Sample image demonstrating the classification of dendritic mushroom, stubby, and thin spines and filopodia in cultured cortical neuron cultures. **(C)** Examples of spine and protrusion shapes and densities after lentiviral infection with shCtrl and shATF4. Culture conditions are as in **(A)**. **(D)** Effect of ATF4 knockdown on the density of different protrusions. Culture conditions and labeling were as in **(A)**. Bar graphs show mean and s.e.m. of 5 individual experiments, *n* = 10 neurons per experiment per condition. ^*^p < 0.05, ^**^p < 0.005. **(E)** Effect of ATF4 knockdown on total spine density. ^**^p < 0.005. **(F)** Effect of ATF4 knockdown on proportions of different protrusions. **(G)** Representative images of hippocampal dendrites after lentiviral infection with shCtrl and shATF4. **(F–J)** Effects of ATF4 knockdown on different protrusions densities **(H)**, proportions **(I)** and total spine density **(J)**. Data represent mean and s.e.m. of 5 individual experiments, *n* = 10 neurons per experiment per condition. ^*^p < 0.05, ^**^p < 0.005.

Although ATF4 is widely expressed in the unstressed brain, its role there is not well-defined. Because ATF4 has been variously implicated in learning and memory, we examined what relevant roles it might play at the cellular level. To do so, we generated lentiviruses expressing either ATF4 shRNA (shATF4-1) or a control construct (shCtrl) in which 5/19 bases in shATF4 were changed. When we infected either cultured rat hippocampal or cortical neurons with shATF4-1 [infection at 7 days *in vitro* (DIV) and assessed at 14 DIV], the knockdown efficiency was over 90% (Figure 1A). Infection with shCtrl, in contrast, had no effect on ATF4 levels compared with no viral treatment (Figure 1A). Immunostaining for eGFP expression also indicated an infection efficiency of more than 90% for both shATF4-1 and shCtrl (Figure 2 and data not shown).

We first used these lentiviruses to examine the effect of ATF4 knockdown on the density of immunostained PSD-95 (post-synaptic density protein 95) dendritic puncta in hippocampal neuron cultures (infection at 6 DIV and assessed at 19 DIV). PSD-95 puncta have been used to indicate excitatory synapse numbers (El-Husseini et al., 2000). Compared with shCtrl, ATF4 knockdown significantly reduced the mean number of PSD-95 puncta/μm of dendritic length by 52 ± 8% (Figures 1B,C). We also tested the effect of ATF4 knockdown on clustering of GluR1, one of the subunits of AMPA receptors that localize to excitatory synapses (Bredt and Nicoll, 2003). Knocking down ATF4 significantly decreased the mean density of GluR1 puncta on dendritic processes by 52 ± 4% (Figures 1D,E). The total levels of PSD-95 and GluR1 in the cultures did not change as assessed by Western immunoblotting (data not shown). These data suggest that, while ATF4 does not alter the total levels of PSD-95 and GluR1, it does regulate the formation of puncta containing these proteins and thus appears to affect post-synaptic development in cultured cortical and hippocampal neurons.

### ATF4 influences spine morphology

Because our data indicate that ATF4 is required for normal numbers of PSD-95 and GluR1 puncta, we next determined whether this might reflect changes in dendritic spine formation and morphology. Cortical neurons cultures were infected with control or shATF4-1 lentiviruses after 5–6 days *in vitro* (when few dendritic spines are present) and analyzed for spine density and spine morphology subtypes at 18 DIV (when spinal maturation has occurred). For the analysis, DiOlistic labeling was used to fill single random neurons in the cultures which were then observed by confocal microscopy (Figure 2A). Infected neurons demonstrated mixed phenotypes of dendritic protrusions which were characterized as either filopodia (long protrusions without head enlargements) or subclassed as mushroom, stubby or thin spines (Figure 2B). Comparison of spine subtype density and distribution in untreated cultures with those treated with control virus showed no significant difference, indicating no non-specific effects of the infection or shRNA construct.

Among the most striking changes seen with ATF4 knockdown was the appearance of long dendritic filopodial protrusions (Figure 2C). There was over a 3-fold increase in the density of such protrusions (Figure 2D) and a 3-fold increase in the proportion of dendritic protrusions that were over 4 μm in length. Examination of the density of individual spine subtypes further revealed that ATF4 knockdown significantly reduced the density of mushroom spines by more than half with no significant effect on stubby or thin spines (Figure 2D). Also, there was a small, but significant decrease in overall spine density (Figure 2E). When the data were analyzed as proportion of total dendritic protrusions, there was a significant decrease in proportion of mushroom and stubby spines and an increase in proportion of filopodia in the ATF4 knockdown neurons (Figure 2F).

Parallel experiments were also carried out on cultured rat hippocampal neurons. Here too, ATF4 knockdown significantly increased the density of filopodia by about 3-fold (Figures 2G,H) and significantly decreased the density of mushroom spines by about half (Figure 2H). As with cortical neurons, there was no significant change in density of stubby or thin spines (Figures 2H,I). Finally, total spine density was not significantly changed by ATF4 knockdown although there was a trend toward a decrease (Figure 2J).

We also assessed whether ATF4 knockdown affected the branching (Sholl analysis) or total lengths of dendrites. At 6 days after knockdown in cortical neurons, there was no change in dendritic branching (Figure S1). In addition, after 12 days and 10 days of knockdown in cortical and hippocampal neurons, respectively, there were not significant alterations in dendrite branching or total dendrite length (Figures S1, S2).

### ATF4 over-expression does not alter spine density or morphology

**Figure 3:**
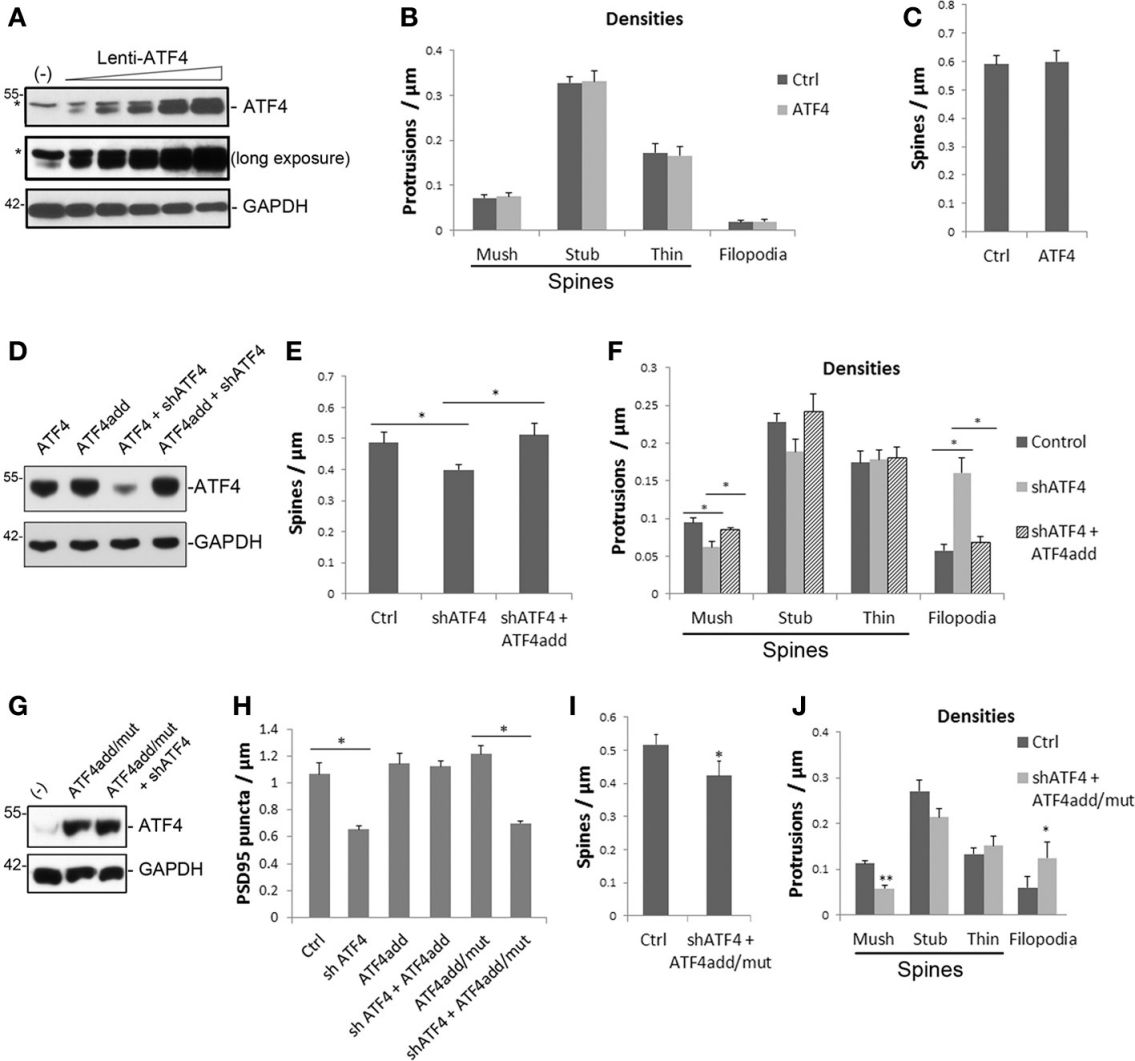
**ATF4 over-expression does not affect total or individual spine densities, while ATF4add, which is not recognized by shATF4, rescues the spine changes caused by ATF4 knockdown and this requires its transcriptional activity. (A)** Overexpression of ATF4 in cultured neurons infected with increasing levels of ATF4 lentivirus at 7 DIV and assessed for ATF4 expression 1 week later. ^*^Indicates a non-specific band on the Western blot. GAPDH serves as loading control. **(B,C)** ATF4 over-expression does not affect total or individual spine densities of cultured hippocampal neurons. Neurons were infected with lenti-ATF4 or lenti-vector at 7 DIV and fixed at 18 DIV and assessed for spine densities. Data represent mean and s.e.m. of 3 individual experiments, *n* = 10 neurons per experiment per condition. **(D)** ATF4add is not recognized by shATF4. HEK293 cells were transfected with lentiviral constructs as indicated. Two days after transfection, total proteins were collected and subjected to Western immunoblotting for overexpressed ATF4. HEK293 cells do not show the non-specific upper band. **(E,F)** ATF4add rescues the changes of total **(E)** and individual spine densities **(F)** caused by knockdown of endogenous ATF4. Cultured cortical neurons were co-infected with lenti-shATF4 plus empty vector or lenti-shATF4 plus lenti-ATF4add and subjected to spine analysis following the time course and procedures described in Figure 2. Data represent mean and s.e.m. of 5 individual experiments, *n* = 10 dendrites per experiment per condition. ^*^p < 0.05. **(G)** ATF4add/mut is not recognized by shATF4. **(H)** ATF4add, but not ATF4add/mut, rescues the change of PSD-95 puncta after ATF4 knockdown. Data represent mean and s.e.m. of 3 individual experiments. ^*^p < 0.05. **(I,J)** ATF4add/mut with a mutation that compromises its DNA binding activity does not rescue the spine density changes caused by ATF4 shRNA. Cultured cortical neurons were co-infected with lenti-shATF4 and lenti-ATF4add/mut. Total spine density is shown in **(I)**. Densities of individual spine subtypes are shown in **(J)**. Data represent mean and s.e.m. of 4 individual experiments. ^*^p < 0.05, ^**^p < 0.005.

To further assess the role of ATF4 in spine formation, we examined whether wtATF4 expression has a reverse effect from ATF4 knockdown in hippocampal neurons. To test this, a lentivirus was generated that expresses wt ATF4 and eGFP (Figure 3A) and this was used to infect hippocampal neuron cultures at 7 DIV. High over-expression of ATF4 (>20-fold) induced signs of neuronal degeneration by 10 days and death after greater than 2 weeks following infection. We therefore used viral titers that elevated overall ATF4 levels by about 5–10 fold; this produced no evident degenerative effects over the course of the experiments. Analysis of spines at 18 DIV revealed no effect on total density or morphology (Figures 3B,C). These findings indicate that while decreasing ATF4 levels affects spine formation in neurons, over-expressing it does not, at least when assessed under the conditions of our study.

### A mutant ATF4 that is resistant to shATF4 rescues changes in spines and PSD95 puncta caused by ATF4 knockdown

To rule out possible off-target effects of the shRNA constructs or lentiviruses used in our experiments, we generated a rescue construct (lenti-ATF4add) that has the same amino acid sequence as wild-type ATF4, but that is mutated at the RNA level so that it is not recognized by shATF4-1 (Figure 3D). Cortical neuron cultures were co-infected with shATF4 and ATF4add lentiviruses at 5–6 days *in vitro* and then analyzed at 18 DIV for spine properties. The presence of ATF4add fully reversed the fall in total spine density (Figure 3E). It also reversed the effect of ATF4 knockdown on filopodia formation and on the density of mushroom spines (Figure 3F). Furthermore, like over-expressed wild-type ATF4, ATF4add did not itself alter spine density or sub-type distribution (Figures 3E,F).

We additionally assessed whether ATF4add would rescue the effect of ATF4 shRNA on PSD-95 puncta in cultures of hippocampal neurons. ATF4add completely reversed the effects of shATF4 on the density of PSD-95 puncta (Figure 3H). As in the case of spine density and morphology, ATF4add alone, or in presence of shATF4, had little or no effect on PSD-95 puncta/μm in our cultures (Figure 3H).

### Regulation of spine morphology and PSD95 puncta by ATF4 requires its transcriptional activity

We next queried whether regulation of spine density and morphology by ATF4 is dependent on its transcriptional activity. To achieve this, we generated an ATF4 lentiviral construct (ATF4add/mut) that was mutated to abolish both its recognition by shATF4 and its DNA-binding capacity. Western blotting confirmed that ATF4add/mut expression is not knocked down by shATF4 (Figure 3G). Cortical neuron cultures were infected with shATF4 and ATF4add/mut as above. This should both knockdown endogenous ATF4 and replace it with an exogenous ATF4 that is transcriptionally inactive. Spine analysis revealed that the transcriptionally inactive ATF4, unlike ATF4add, failed to reverse the effects of ATF4 knockdown on total spine density and on the densities of mushroom spines and filopodia (Figures 3I,J). Parallel experiments were carried out on hippocampal cultures which were assessed for PSD-95 puncta. Here also, in contrast to ATF4add, ATF4add/mut failed to reverse the effects of ATF4 knockdown (Figure 3H). Thus, it appears that regulation of spine density and morphology as well as PSD-95 puncta by ATF4 requires its transcriptional activity.

### Knocking down ATF4 down-regulates total RhoA and Cdc42 levels in cultured neurons, but not Rac1

**Figure 4:**
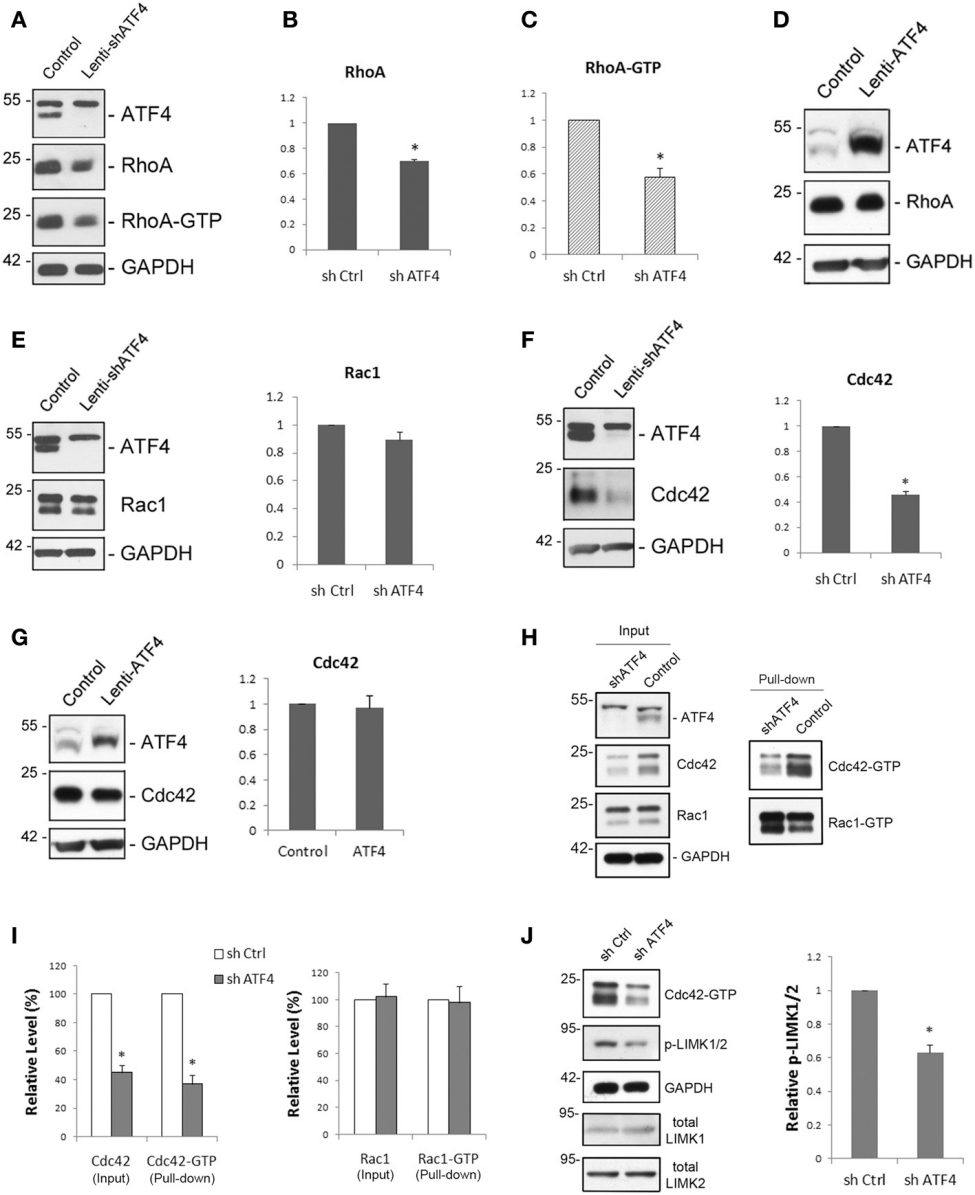
**Knockdown of ATF4 down-regulates total levels of RhoA and Cdc42, but not of Rac1. (A–C)** ATF4 knockdown reduces RhoA and RhoA-GTP levels. Cultured cortical neurons were infected with lenti-shATF4 or lenti-Control and total cell lysates were collected at different time points. Western immunoblotting **(A)** shows the decrease of total RhoA and RhoA-GTP after 8 days of infection. Quantifications of total RhoA and RhoA-GTP levels after 12 days of lentiviral infection are shown in **(B)** and **(C)**, respectively. Data represent mean and s.e.m. of 4 individual experiments. ^**^p < 0.005. **(D)** Overexpression of ATF4 in cortical neurons by lentivirus does not change total RhoA levels. **(E)** ATF4 knockdown has no effect on Rac1 levels. Cortical neurons were cultured and analyzed as in **(A)**. **(F)** ATF4 knockdown reduces Cdc42 protein expression. Bar graphs show mean and s.e.m. of 4 individual experiments. ^**^p < 0.005. **(G)** Overexpression of ATF4 in cortical neurons by lentivirus does not change total Cdc42 levels. **(H,I)** ATF4 knockdown also decreases Cdc42-GTP, but not Rac1-GTP. Cultured cortical neurons (10 days infection) were collected and subjected to immunoprecipitation by using PAK-GST beads which bind to the GTP-bound Rac and Cdc42. Cdc42-GTP and Rac1-GTP were detected by Western immunoblotting with anti-Cdc42 and anti-Rac **(H)**. Quantifications are shown in **(I)**. Data represent mean and s.e.m. of 3 individual experiments. ^*^p < 0.05. **(J)** Knocking down of ATF4 decreases the levels of phospho-LIMK1/2. Experimental conditions are as in **(H)**. Data represent mean and s.e.m. of 3 individual experiments. ^*^p < 0.05.

Because spine formation and morphology are highly sensitive to the status of the actin cytoskeleton, we examined whether loss of ATF4 might affect expression of actin-regulatory proteins. In particular, we focused on the small GTPases RhoA, Rac1, and Cdc42 that regulate actin cytoskeletal assembly and stability in dendrites and thereby affect spine shape and formation (Newey et al., 2005). We first infected cortical cultures at 7 DIV with control and shATF4 lentiviruses and examined the levels of RhoA-GTP (the active form of the protein) as well as of total RhoA over time. A decrease in both RhoA-GTP and total RhoA proteins was evident by 8 days following ATF4 knockdown (Figure 4A) and this reached a level of 30 ± 2% loss for total RhoA and 42 ± 6% for RhoA-GTP by 12 days relative to control (Figures 4B,C). Thus, it appears that ATF4 regulates total RhoA expression in cortical neurons and that this is reflected by a comparable change in the active form, RhoA-GTP. We also assessed the effect of ATF4 over-expression on RhoA protein levels to determine if it had the opposite effect of ATF4 knockdown. However, elevating ATF4 in cortical neurons by lentiviral infection did not increase levels of RhoA (Figure 4D) or RhoA-GTP (data not shown).

In addition to RhoA, we examined the role of ATF4 in regulating neuronal levels of Rac1 and Cdc42 proteins. There was no significant effect on total Rac1 protein levels in cultured cortical and hippocampal neurons after lentiviral knockdown of ATF4 (Figure 4E and data not shown). In contrast, infection with shATF4 lentivirus caused 54 ± 3% (Figure 4F) and 56 ± 11% decreases in total Cdc42 protein levels in cortical and hippocampal neurons, respectively. There was no effect of ATF4 over-expression on Cdc42 protein levels (Figure 4G). Furthermore, when we assessed the levels of Cdc42-GTP and Rac1-GTP using PAK-GST beads which bind the active GTP-bound forms of these proteins, no change in Rac1-GTP level was observed while there was a 63 ± 6% decrease in Cdc42-GTP levels (Figures 4H,I). To verify that the change of total Cdc42 affects its downstream targets, we also examined the phosphorylation level of LIMK1/2, effectors of Cdc42 that are involved in actin polymerization (Meng et al., 2002). Knockdown of ATF4 in cortical neurons resulted in a 37 ± 5% decrease in phospho-LIMK1/2 (Figure 4J). Thus, taken together, these findings indicate that ATF4 is required for maintaining levels of total RhoA and Cdc42 proteins and of their corresponding activated forms in neurons and that elevation of ATF4 alone has no effect on these proteins.

### Knockdown of Cdc42, but not RhoA affects spine morphology

**Figure 5:**
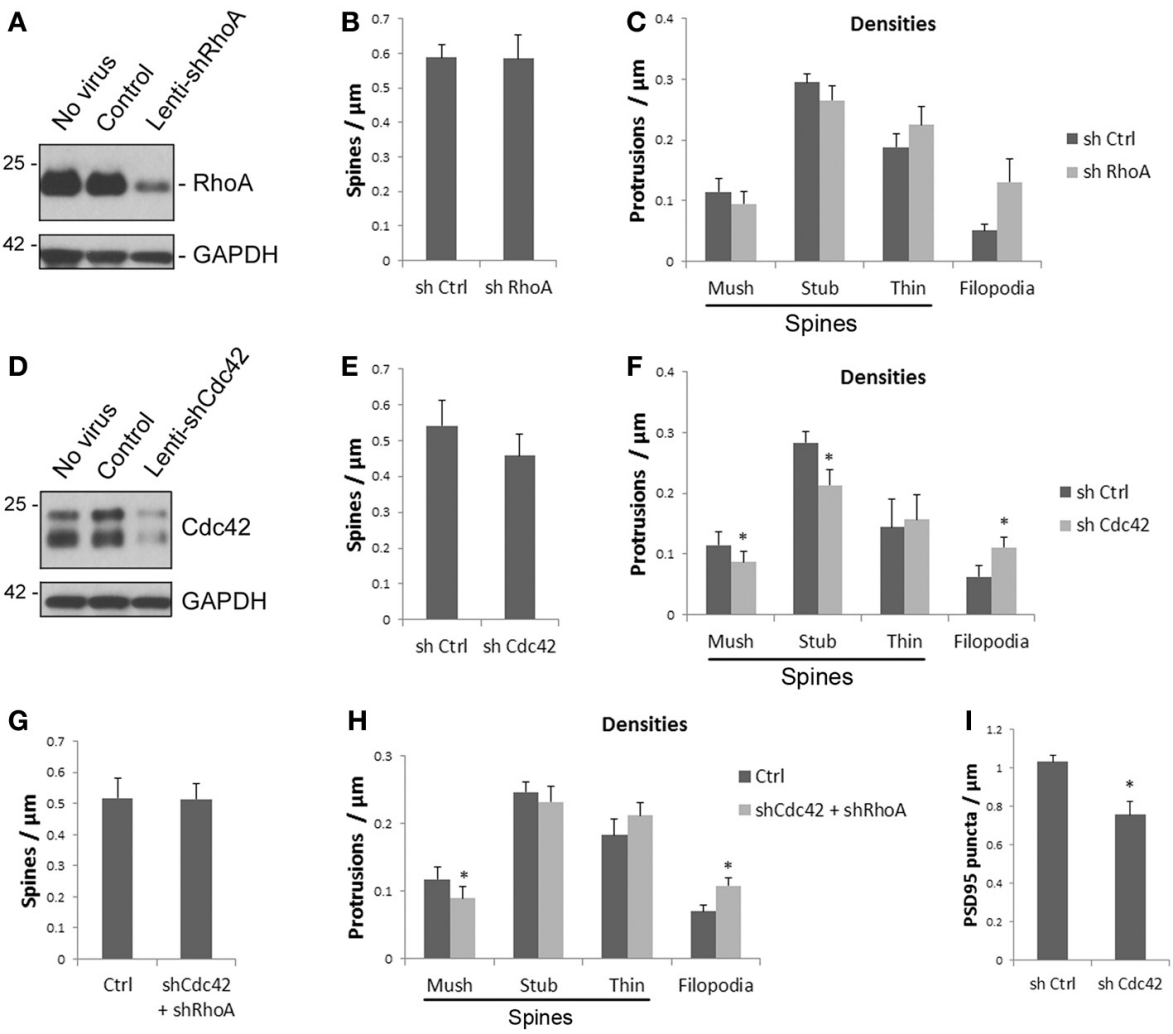
**Knockdown of Cdc42, but not RhoA affects spine morphology and PSD95 puncta density. (A)** Lenti-shRhoA efficiently knocks down endogenous RhoA protein. Cultured cortical neurons were infected with lentiviruses as indicated at 7 DIV. Total proteins were harvested at 14 DIV and subjected to Western immunoblotting. **(B,C)** Knockdown of RhoA does not affect spine densities. Cultured cortical neurons were infected with lenti-shRhoAat 6 DIV and analyzed at 18 DIV. Knockdown of RhoA does not significantly affect total spine density **(B)** and the densities of individual spine subtypes **(C)**. Data represent mean and s.e.m. of 5 individual experiments. **(D,E)** Knockdown of Cdc42 significantly affects the densities of individual spine subtypes. Lenti-shCdc42 efficiently knocks down the endogenous Cdc42 protein. Both bands are Cdc42 protein **(D)**. Total spine density **(E)** and the densities of individual spine subtypes **(F)** were analyzed as in **(B)**. Data represent mean and s.e.m. of 4 individual experiments. ^*^p < 0.05. **(G)** Simultaneous knockdown of RhoA and Cdc42 has no effect on total spine density. Cultured cortical neurons were co-infected with lenti-shRhoA and lenti-shCdc42. Analysis was performed at the same time point as above. Bar graphs show mean and s.e.m. of 3 individual experiments with cortical neurons carried out as above. **(H)** Effect of simultaneously knocking down both RhoA and Cdc42 on densities of individual spine subtypes. Data represent mean and s.e.m. of 5 individual experiments. ^*^p < 0.05. **(I)** Knockdown of Cdc42 decreases the density of dendritic PSD95 puncta. Data represent mean and s.e.m. of 3 individual experiments. ^*^p < 0.05.

To determine whether the reductions in RhoA and Cdc42 proteins observed after ATF4 knockdown may contribute to the effects of shATF4 on spine density and shape, we generated lentivirus expressing RhoA- and Cdc42-shRNAs. Cortical neurons were infected with the viruses at day 7 *in vitro* and Western immunoblotting 8 days later showed reduction of endogenous RhoA and Cdc42 levels by over 80 (Figure 5A) and 70% (Figure 5D), respectively. Analysis of cultured cortical neurons (on day 18 *in vitro* after infection on day 6) indicated that knockdown of RhoA caused no significant change in total spine density (Figure 5B) or in densities of individual spine subtypes, although there was a trend toward an increase in filopodial-like protrusions (*p* = 0.08; *n* = 5 independent experiments) (Figure 5C). In contrast, Cdc42 knockdown resulted in a significant increase in formation of filopodia and decreases in the densities of both mushroom and stubby spines (Figures 5E,F). There was also a trend toward a decrease in total spine density.

The above findings indicate that ATF4 shRNA reduces both RhoA and Cdc42 protein expression, and that knockdown of Cdc42, but not RhoA, significantly reduces mushroom and stubby spine densities and significantly increases the density of filopodia. The effect of Cdc42 knockdown was qualitatively similar to that achieved by ATF4 knockdown, but was less robust and did not significantly affect overall spine density. We therefore examined whether simultaneously knocking down both Cdc42 and RhoA would result in a stronger effect on spines. However, the effect of the combined knockdown on mushroom spines and filopodia was similar to that seen with Cdc42 knockdown alone, with no significant change in density of stubby spines or of overall spine density (Figures 5G,H). Thus, it appears that while ATF4 influences spines in part via regulation of Cdc42, other yet unidentified molecules also contribute to this effect.

### Knockdown of Cdc42 reduces PSD95 puncta

We next asked whether the effects of ATF4 on Cdc42 levels may also underlie the reduction in PSD-95 puncta seen when ATF4 is silenced. If this is the case, then Cdc42 knockdown should evoke similar decreases in puncta density as obtained by silencing ATF4. Compared with control shRNA, infection with Cdc42 shRNA reduced the density of PSD-95 puncta in cultures of hippocampal neurons by 24 ± 7% (Figure 5I). These findings indicate that regulation of Cdc42 levels contributes at least in part to the effects of ATF4 on the densities of synaptic puncta as well as dendritic spines.

### ATF4 knockdown affects the stability of Cdc42

**Figure 6:**
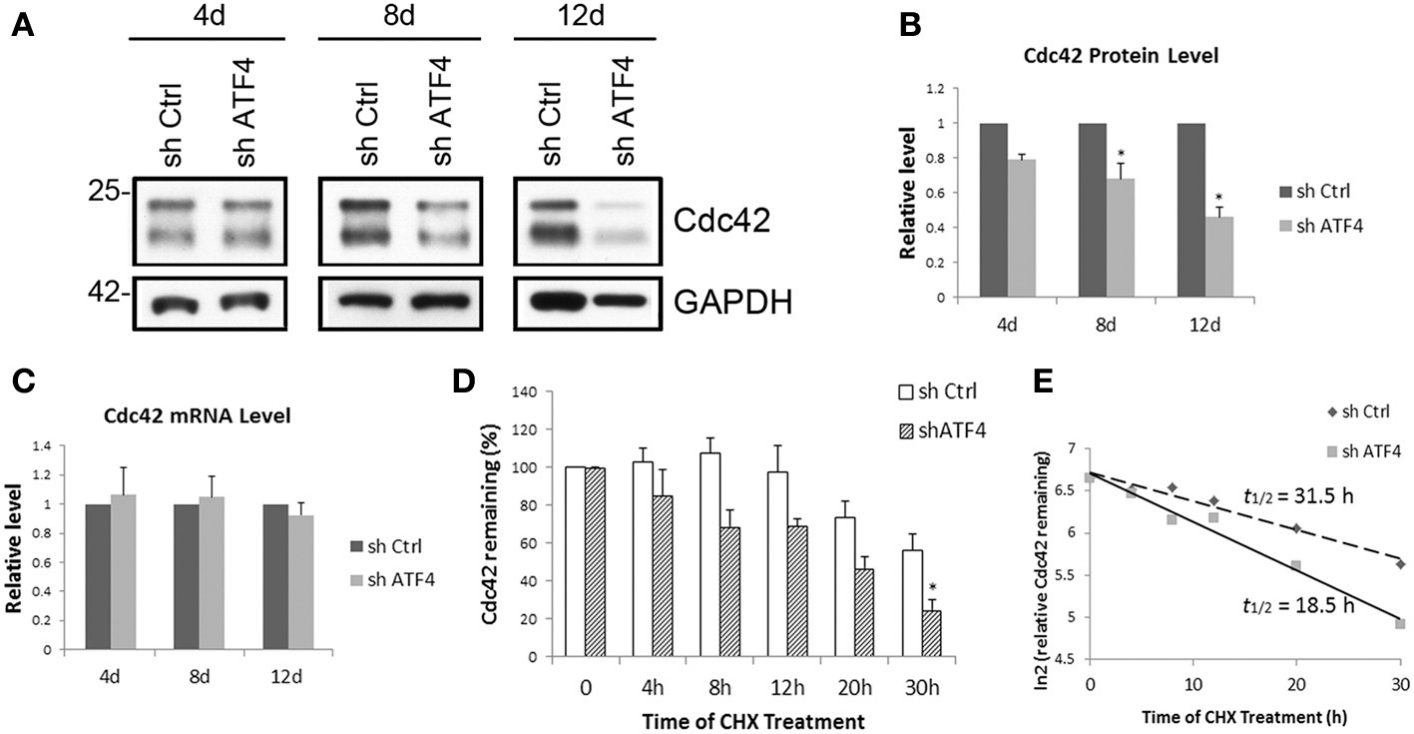
**ATF4 knockdown decreases the half-life of Cdc42 in cortical neurons. (A)** Time course of the effect of ATF4 knockdown on Cdc42 protein levels. Primary cortical neurons were infected with lenti-shCtrl and shATF4 at 6 DIV. After 4, 8, and 12 days infection, cells were harvested and subjected to Western immunoblotting. **(B)** Quantification of Cdc42 protein level relative to control. Data represent mean and s.e.m. of 3 individual experiments. ^*^p < 0.05. **(C)** ATF4 knockdown does not affect Cdc42 mRNA levels. Total RNA from cultured cortical neurons was collected at the time points as **(A)** and subjected to quantitative real time PCR to detect Cdc42 message levels. Data represent mean and s.e.m. of 3 individual experiments. **(D,E)** ATF4 knockdown decreases the half-life of Cdc42 protein in cortical neurons. Replicate cortical neuron cultures were infected with lentivirus expressing either shCtrl or shATF4 after 5–7 days *in vitro*. Seven days later the cultures were treated with 10 μM cycloheximide for the indicated times and then analyzed for Cdc42 by Western blotting analysis **(D)**. The relative levels of Cdc42 remaining after the indicated times (0 time = 100) were expressed on a ln2 scale **(E)**. Data are means of four independent experiments. Straight lines were fitted by least-squares analysis (*r*^2^ = 0.96 for control; 0.98 for shATF4). The calculated half-lives were 31.5 h for control and 18.5 h for cells infected with shATF4.

Because the action of ATF4 in regulating spine morphology is mediated at least in part by Cdc42, we further investigated the mechanism by which ATF4 influences neuronal Cdc42 protein expression. To assess whether the effect of ATF4 knockdown on Cdc42 occurs at the transcriptional level, we measured Cdc42 transcript levels by q-PCR at various times after infection with control and shATF4 lentivirus. While Cdc42 protein decreased along with the duration of infection (Figures 6A,B), there was no significant change in Cdc42 mRNA levels in cortical (Figure 6C) or hippocampal (data not shown) neurons after ATF4 knockdown. These findings thus indicate that the effects of ATF4 on Cdc42 protein are not due to regulation of Cdc42 transcript levels.

We next assessed whether ATF4 might regulate the post-transcriptional stability of Cdc42 protein. To achieve this, cultured cortical neurons with or without lentiviral ATF4 knockdown were treated with cycloheximide to block protein translation and assessed after various times for Cdc42 contents. This revealed that knockdown of ATF4 accelerated Cdc42 turnover (Figure 6D) and that the half-life of Cdc42 protein was reduced by ATF4 knockdown from 31.5 to 18.5 h (Figure 6E). Thus, ATF4 is required for stabilization of Cdc42 protein and ATF4 knockdown reduces Cdc42 protein half-life, which in turn influences mushroom spine and filopodia formation as well as the density of synaptic puncta.

### Knockdown of ATF4 in the adult hippocampus decreases the proportion of mushroom spines

**Figure 7:**
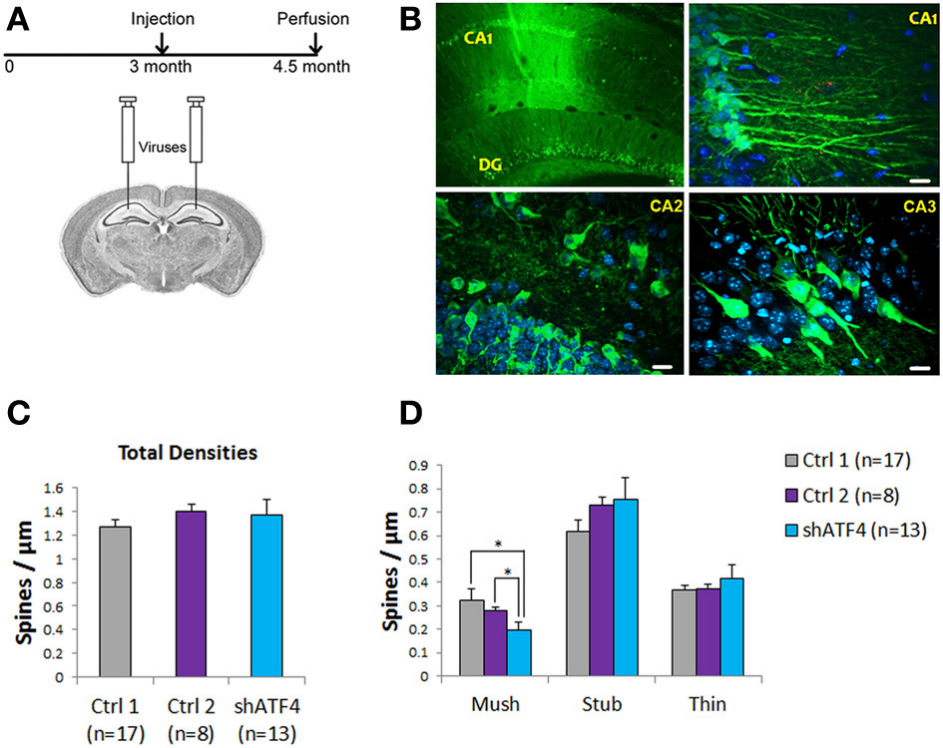
**Knockdown of ATF4 *in vivo* decreases the density of hippocampal mushroom spines. (A)** Schematic representation shows the time course for injection and perfusion in this experiment. **(B)** Images of infected cells (green, GFP-positive) in mouse brain sections. After perfusion, mouse brains were sectioned and immunostained (anti-GFP) to visualize infected cells. Low magnification (10×) image shows the CA1 region and dentate gyrus (left); high magnification (40×) image shows neurons and processes in the CA1–CA3 regions (right). Scale bar, 20 μm. **(C)** Total spine density of the infected neurons from injected mice. Dendrites from 10 infected neurons per animal (*n* = number of animals) were randomly scored per animal. **(D)** Densities of different spine subtypes in hippocampi infected with control 1, control 2, and ATF4 shRNAs. Dendritic spines were scored as mushroom, stubby and thin subtypes. Data represent mean and s.e.m. ^*^p < 0.05.

To determine whether knockdown of ATF4 would also affect spines *in vivo*, we stereotactically injected ATF4-shRNA-1 lentivirus (which also co-expresses eGFP) into the hippocampi of 3 month-old mice (Figure 7A). To rule out potential non-specific effects of the shRNAs or of the viral infection, we also injected animals with two control lentiviral constructs (control 1, a scrambled form of ATF4-shRNA and control 2, an ineffective ATF4 shRNA targeted to the rat ATF4 sequence but not the mouse ATF4 message). To judge the efficacy of the lentiviral infection and to identify infected cells, we assessed eGFP expression 6 weeks later. There was wide-spread expression in the CA1 region and dentate gyrus as well as in the CA2 and CA3 regions (Figure 7B). Neuron counts indicated an infection efficiency of about 50%. Six weeks after infection, hippocampi were harvested from shControl-1/2 and shATF4 animals and single random neurons in hippocampal slices were stained with DiI by DiOlistic labeling. Evaluation of dendritic spines in infected (GFP+) cells revealed no significant differences in overall spine density (Figure 7C) or in mean densities of stubby or thin spines; no filopodia were present under any condition (Figure 7D). However, as found *in vitro*, there was a significant decrease in the density of mushroom spines by 39 ± 9% in neurons infected with shATF4 compared with control 1 and 29 ± 11% compared with control 2 (Figure 7D). Thus, in the adult mouse hippocampus, knockdown of ATF4 reduces the density of mushroom spines as it does *in vitro*, and does not appear to affect other spine types.

## Discussion

Despite ATF4's ubiquitous presence in brain and its implication as either a positive or negative regulator of learning and memory, little is known about its effects on neurons at the cellular level and how these in turn might affect function. Part of the ambiguity regarding ATF4's function arises from the use of reagents that act on its expression by indirect and likely non-selective means. Also, ATF4 null mice suffer from a variety of defects including anemia, bone dysgenesis, and micropthalmia that complicate experimental design and interpretation. Here, we have used lentiviral-based knockdown and addback/rescue strategies to probe the role of ATF4 in neurons at the cellular level. We have focused on ATF4's role in synapse formation and dendritic spine morphology and on the mechanism by which it affects these features.

Our study examined the effects of ATF4 manipulation on PSD-95 and GluR1 puncta, which are post-synaptic markers often considered indicative of excitatory synaptic contacts. Our experiments show that ATF4 knockdown reduced the density of dendritic PSD-95 and GluR1 puncta in cultures of hippocampal neurons by 40–50%. While these markers are post-synaptic, it is likely that they reflect overall synaptic density. These findings thus suggest that ATF4 plays an important and required role in synapse formation and that in its absence, connectivity is submaximal. We also observed that ATF4 knockdown consistently reduces the density of dendritic mushroom spines by 40–50% in cultured cortical and hippocampal neurons and by about 30–40% in hippocampal neurons *in vivo*. Mushroom spines have been considered to be the most mature and stable of spine forms and it has been suggested that they may serve as “memory spines” with enhanced excitatory synaptic transmission compared with other spine subtypes (Bourne and Harris, 2007; von Bohlen Und Halbach, 2009). Thus, the effects of ATF4 shRNA on spines are further consistent with a role for ATF4 as a positive regulator of synapse formation.

Because the actin cytoskeleton plays a major role in spine formation and morphology as well as in synapse formation (Hotulainen and Hoogenraad, 2010; Bosch and Hayashi, 2012), we examined whether effects on actin-regulatory Rho family small GTPases might underlie the mechanism by which ATF4 affects neurons. We found that the total levels of neuronal RhoA/RhoA-GTP and of Cdc42/Cdc42-GTP, but not of Rac1, decrease by nearly half after ATF4 knockdown. The response of Cdc42 in particular appeared to be relevant since shRNA-promoted knockdown of Cdc42 qualitatively reproduced the effect of ATF4 silencing on PSD95 puncta and on mushroom spines and filopodia. In addition, ATF4 silencing significantly reduced the phosphorylation of Cdc42 effectors LIMK1/2. Taken together, these results indicate that ATF4 is required for maintaining normal neuronal levels of active and total Cdc42 and that the reduction in these proteins that occurs after ATF4 knockdown mediates, at least in part, the consequent fall in excitatory synapse density, decrease in density of dendritic spines and increase in long dendritic filopodial processes. These ideas are consistent with reports that mice deficient in LIMK-1, a downstream effector of Cdc42, or that neuronally overexpress a dominant-negative form of Cdc42, have a reduced density of mushroom spines (Meng et al., 2002; Vadodaria et al., 2013). Although there was a trend, RhoA knockdown did not significantly reduce mushroom spine density. Nor did silencing of both RhoA and Cdc42 produce a greater effect than knocking down Cdc42 alone. Thus, the consequences of the effects of ATF4 on RhoA levels remain to be seen.

We further probed the mechanism by which ATF4 affects Cdc42 levels in neurons. Although neuronal Cdc42 protein levels fall in response to ATF4 silencing, there were no changes in the levels of its corresponding mRNA. In contrast, we found that the half-life of Cdc42 in neuronal cultures was reduced by about 40% when ATF4 was silenced, suggesting that ATF4 regulates the stability rather than synthesis of this protein. On the other hand, our rescue experiments indicated that ATF4 must retain transcriptional competence to influence spine morphology and synaptic density. Thus, it appears that ATF4 influences the levels of Cdc42 by a transcriptional mechanism that in turn indirectly leads to changes in its stability. Currently, no E3 ligase for Cdc42 has been reported. The E3 ligase Smurf1 is reported to promote degradation of RhoA, but we found no effect of ATF4 knockdown on expression of this protein. We also carried out a chip-based transcriptome analysis of cultured cortical neurons infected with control or sh-ATF4 lentiviruses. Although we found changes in a number of previously described ATF4 targets, there were no changes in genes with clearly-defined potential roles in regulation of Cdc42 stability (data not shown). Further studies are therefore needed in order to uncover how ATF4 regulates the turnover of Cdc42.

In addition to affecting mushroom spines in cultured neurons, ATF4 silencing markedly increased the density and lengths of dendritic filopodia. These are generally considered to be developmentally transient structures that can transform into spines or disappear (Fiala et al., 1998). In line with this, we saw no effect on these structures when ATF4 was silenced in adult hippocampal neurons *in vivo*. While rare on mature neurons, abnormally long dendritic filopodia similar to those observed here in culture have been reported in pathological situations such as mental retardation in which they appear to exist at the expense of mushroom and stubby spines (Purpura, 1974). Formation of dendritic filopodia in response to ATF4 knockdown appeared to be mediated at least in part by loss of Cdc42 in that knockdown of this protein resulted in an increased density of these protrusions.

In contrast to the effects of ATF4 down-regulation on synapses, spines and Rho family members, there were no effects of ATF4 over-expression on these parameters. This could reflect that endogenous ATF4 levels in our systems were already at maximal levels with regard to synapse, spine and Rho family regulation. Alternatively, ATF4 may require binding partners for these actions that are present in limiting amounts. Additionally, there are also feed-back mechanisms, such as ATF4-induced miRNAs, that may serve to dampen cellular responses to ATF4 over-expression (Chitnis et al., 2012) in order to keep the system at optimal activity.

As a basic leucine zipper family member, ATF4 has the capacity to bind a variety of other proteins such as GABAB receptors and DISC1 protein that might affect spines by non-transcriptional mechanisms (Nehring et al., 2000; Morris et al., 2003). In this regard, it is significant that our rescue experiments demonstrate that ATF4 requires transcriptional activity to regulate spines and excitatory synapses. ATF4 thus joins a group of transcription factors including FoxO6, Mef2, NFkB, MeCP2, and CREB that regulate dendritic spine density and/or morphology (Murphy and Segal, 1997; Fukuda et al., 2005; Flavell et al., 2006; Russo et al., 2009; Salih et al., 2012).

In summary, our studies establish that the transcription factor ATF4 plays a required role in regulating synapse formation and morphology of dendritic spines. These actions appear to be mediated at least in part by an indirect effect on Cdc42 stability and levels. These findings provide a potential cellular basis for the reported contributions of ATF4 to plasticity and memory.

### Conflict of interest statement

The authors declare that the research was conducted in the absence of any commercial or financial relationships that could be construed as a potential conflict of interest.

## Supplementary material

Figure S1 **ATF4 knockdown doesn't affect the total dendrite length and dendritic complexity of cultured cortical neurons. (A)** Immunoblotting shows the knockdown efficiency of shATF4-1 and shATF4-9. Primary cortical neurons were infected with indicated lentiviruses on 5 DIV and harvested on 12 DIV. GAPDH served as loading control. **(B)** Sample images show the dendritic complexity. Rat E18 primary cortical neurons were infected with viruses after 2 DIV and fixed on 14 DIV. Neurons were DiOlistically labeled and images were captured under 20× magnification. Scale bar = 50 μm. **(C)** ATF4 knockdown doesn't alter the total dendrite length of infected neurons at14 DIV. Eight to ten GFP-positive and Dil labeled neurons were evaluated by the NeuronJ plug-in in ImageJ (NIH). **(D)** ATF4 knockdown has no effect on dendritic complexity of cortical neuron on 14 DIV. Eight to ten neurons were evaluated using the Shollanalysis plug-in in ImageJ. Data represent mean and s.e.m.

Figure S2 **ATF4 knockdown has no effect on total dendrite length and dendritic complexity of cultured hippocampal neurons. (A)** Knockdown of ATF4 does not change the total dendrite length of infected neurons. Rat E18 primary hippocampal neurons were infected with control or shATF4-1 virus after 2 DIV and fixed on 12 DIV. Neurons were DiOlistically labeled and images were captured under 20× magnification. **(B)** Knockdown of ATF4 has no effect on dendritic complexity of hippocampal neuron on 12 DIV. Data was analyzed as in Figure S1.
