# Supplementary figures and images for: Activating transcription factor 4 (ATF4) modulates post-synaptic development and dendritic spine morphology
Source: Front Cell Neurosci. 2014 Jun 30;8:177. doi: 10.3389/fncel.2014.00177 (PMC4075335; doi:10.3389/fncel.2014.00177)

S1

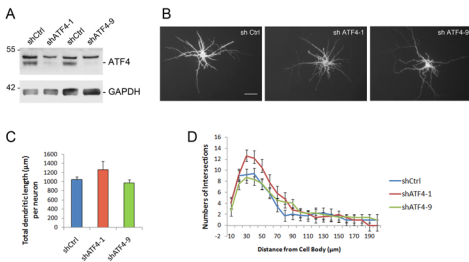

S2

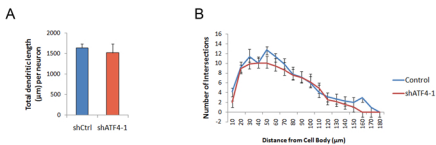

Supplement: Figure S1 — ATF4 knockdown doesn't affect the total dendrite length and dendritic complexity of cultured cortical neurons. (A) Immunoblotting shows the knockdown efficiency of shATF4-1 and shATF4-9. Primary cortical neurons were infected with indicated lentiviruses on 5 DIV and harvested on 12 DIV. GAPDH served as loading control. (B) Sample images show the dendritic complexity. Rat E18 primary cortical neurons were infected with viruses after 2 DIV and fixed on 14 DIV. Neurons were DiOlistically labeled and images were captured under 20× magnification. Scale bar = 50 μm. (C) ATF4 knockdown doesn't alter the total dendrite length of infected neurons at14 DIV. Eight to ten GFP-positive and Dil labeled neurons were evaluated by the NeuronJ plug-in in ImageJ (NIH). (D) ATF4 knockdown has no effect on dendritic complexity of cortical neuron on 14 DIV. Eight to ten neurons were evaluated using the Shollanalysis plug-in in ImageJ. Data represent mean and s.e.m. [file Presentation1.PDF]
